# Identification of SNP loci and candidate genes related to four important fatty acid composition in *Brassica napus* using genome wide association study

**DOI:** 10.1371/journal.pone.0221578

**Published:** 2019-08-23

**Authors:** Qianglong Zhu, Graham J. King, Xingyue Liu, Nan Shan, Priyakshee Borpatragohain, Abdul Baten, Putao Wang, Sha Luo, Qinghong Zhou

**Affiliations:** 1 Key Laboratory of Crop Physiology, Ecology and Genetic Breeding, Ministry of Education, Agronomy College, Jiangxi Agricultural University, Nanchang, China; 2 Southern Cross Plant Science, Southern Cross University, Lismore, NSW, Australia; Huazhong University of Science and Technology, CHINA

## Abstract

Rapeseed oil (canola, *Brassica napus* L.) is an important healthy vegetable oil throughout the world, the nutritional and economical value of which largely depends on its seed fatty acid composition. In this study, based on 201,187 SNP markers developed from the SLAF-seq (specific locus amplified fragment sequencing), a genome wide association study of four important fatty acid content traits (erucic acid, oleic acid, linoleic acid and linolenic acid) in a panel of 300 inbred lines of rapeseed in two environments (JXAU and JXRIS) was carried out. A total of 148 SNP loci significantly associated with these traits were detected by MLM model analysis respectively, and 30 SNP loci on A08 and C03 chromosomes were detected in three traits of erucic acid, oleic acid and linoleic acid contents simultaneously. Furthermore, 108 highly favorable alleles for increasing oleic acid and linoleic acid content, also for decreasing erucic acid content simultaneously were observed. By a basic local alignment search tool (BLAST) search with in a distance of 100 Kb around these significantly SNP-trait associations, we identified 20 orthologs of the functional candidate genes related to fatty acid biosynthesis, including the known vital fatty acid biosynthesis genes of *BnaA*.*FAE1* and *BnaC*. *FAE1* on the A08 and C03 chromosomes, and other potential candidate genes involving in the fatty acid biosynthesis pathway, such as the orthologs genes of *FAD2*, *LACS09*, *KCS17*, *CER4*, *TT16* and *ACBP5*. This study lays a basis for uncovering the genetic variations and the improvement of fatty acid composition in *B*. *napus*.

## Introduction

Canola (rapeseed; *Brassica napus*, AACC genome, 2n = 38) is the world’s second largest oil producing crop after soybean (*Glycine max* L. Merrill), cultivated in temperate regions of many countries worldwide, and accounts for 14% of all edible vegetable oil production [1]. The nutritional and healthy oil qualities of canola seed are mostly determined by the fatty acid compositions [2]. Canola oil is rich in unsaturated fatty acids compared with other vegetable oils, comprised primarily of monounsaturated oleic acid and polyunsaturated linoleic and linolenic acid having a 2:1 optimal ratio [3, 4]. Of these the oleic acid (C18:1) and linoleic acid (C18:2) are considered to be healthy and nutritious. However, the three double-bonds of extracted linolenic acid (C18:3) are easily oxidized, which leads to a reduced frying thermal stability and storage time of the oil. With erucic acid originally thought to lead to health problems and difficult to digest in humans and livestock due to its long-chain, modern canola-type rapeseed was selected based on ‘double-low’ seed erucic acid and glucosinolate content [5], Reducing erucic acid and linolenic acid has continued to be an important target for canola/rapeseed production [6].

The genetic basis of seed fatty acid biosynthesis and modification pathways have been well characterised in *Arabidopsis thaliana* [7–9]. Barker *et al* (2007) established desaturation and elongation pathways well, along with substrate: product relationships assigned to specific enzymes [10]. To better understand the genetic control of seed fatty composition and biosynthesis in rapeseed, in the last few decades, many underlying QTLs of seed quality traits have been detected in bi-parental segregating populations. These QTL have included traits such as oil content [11–14], protein content [15, 16], glucosinolate content [17, 18], and fatty acid composition [19–21]. In a number of cases, some candidate genes have been identified that coincide with the position of QTL. For example, four loci for *B*. *napus* orthologues of *FAD2* (*BnaFAD2* loci) were mapped to A1, A5, C1 and C5 chromosomes [22], Schierholt *et al*. (2000) also mapped a locus linked to *BnaFAD2* on A05 [23], and Hu *et al*. (2006) identify a major locus for high oleic acid (C18:1) on A5 chromosome [3], which was proven to be the *Fatty acid desaturase-2* (*FAD2*) gene. In addition, two important *FAE1* loci on chromosome A08 and C03 were described by Qiu *et al*. (2006) [11].

In recent years, genome wide association study (GWAS), also known as association mapping based on linkage disequilibrium, have aimed to identify genetic variants linked to traits. GWAS uncover QTLs or genes from natural populations, and have the advantage of higher resolution and greater cost-effectiveness relative due to screening a larger effective number of recombination events than are accessible in moderate size bi-parental segregating populations. GWAS has successfully been demonstrated to be a powerful tool for dissecting complex traits for crop improvement programs, with the availability of numerous SNPs it has been applied to *Zea mays*, *Triticum aestivum* and *Oryza sativa* [24]. In recent years, many studies have used the Illumina Infinium Brassica 60K SNP array and Dart-seq genotyping approaches to carry out GWAS to detect genetic variation for flowering time, as well as seed quality traits in rapeseed [25–33]. The number of robust and well-distributed SNP markers across genome is significant for the efficiency of GWAS in wider germplasm sets. Development of next generation sequencing (NGS) allows identification of a large number of genetic makers for associating with traits of interest based on linkage disequilibrium quickly and efficiently [34]. To identify novel loci and candidate genes that may contribute to variation in fatty acid composition, we carried out an extensive GWAS based on 201,817 SNPs previously developed by SLAF-seq (specific length amplified fragment sequencing) [35,36] and a collection of 300 inbred rapeseed lines. Interactions between the traits were investigated and significantly associated SNP loci were explored along with candidate genes. Favourable allelic variants contributing to an optimal fatty acid composition were identified. This study provides useful information for a more comprehensive understanding of the genetic variation and metabolism mechanism of important fatty acid composition.

## Materials and methods

### Plant materials, growth conditions and field trials

A world-wide collection of 300 diverse rapeseed inbred lines (S4 generation or greater) (S1 Table) was assembled. The provenance and meta-data and genetic relationships for all accessions have been described previously [35,36]. The association population was planted and harvested in the crop field of Jiangxi Agricultural University (JXAU, 115.84E, 28.77N) and Jiangxi Institute of Red Soil (JXIRS, 116.27E, 28.37N) with two biological replications per experimental site in 2014–2015. All seeds were sown on September 29^th^ 2014 simultaneously in both places. Each variety was planted in a plot with three rows (40 cm line width and 20 cm plant distance), and each row had 12 plants (final seeding time was at the 5–7 leaf phase). Field experiments were arranged and laid out in a randomized complete block design at all sites. All rapeseed inbred lines grown in both environments was cultivated under uniform agronomic practices. Ripe seeds from six plants each accession were harvested and used for seed quality trait analysis after harvests.

### Fatty acid compositions evaluation and statistical analysis

The harvested seeds (2g each accession) of 300 rapeseed lines were analysed for estimates of the four fatty acids (erucic acid, oleic acid, linoleic acid and linolenic acid) (%) using DA7200 near infrared spectroscopy (NIRS) (DA 7200, Perten Instruments, Huddinge, Sweden), and the data of four fatty acid content from NIRS were adjusted by the results of gas chromatograph. The value of each fatty acid was expressed as a percentage of the total amount of fatty acids identified. The four fatty acid composition traits of each accession were defined as the mean of the two replicates in the same location. The correlation coefficients between each pair of traits were determined using Student’s t-test, and the variance and statistical analysis of four components were obtained using DPS software [37].

### SNP genotyping and population structure analysis

Total genomic DNA was extracted from young leave of each rapeseed accession using a modified cetyltrimethylammonium bromide (CTAB) method based on Murray & Thompson (1980) [38], the DNA concentrations and purities of all samples were calculated by a Nanodrop 2000 UV-Vis spectrophotometer (NanoDrop, Wilmington, DE, USA). Quantified DNA samples were used for SLAF sequencing by an Illumina Hiseq^TM^ 2500 [39]. The library construction, paired-end sequencing and SNP calling were conducted as previously described [35,36], a total of 201,817 SNPs with minor allele frequency (MAF) > 0.05 and integrity > 0.8 were selected and used for subsequent analysis, population structure (Q matrix) and relative kinship (K matrix) were analysed by using the Admixture software package [40] and SPAGeDi software [41], respectively, as previously described in our previous research [35,36].

### Genome wide association analysis

Based on the 201,817 SNP markers developed for the 300 rapeseed accessions, genome-wide association analysis for the four fatty acid traits was carried out by mixed linear models (MLM) using the Tassel 5.0 software [42]. Fixed effects and random effects in the MLM model were assessed by a Q and K matrix, respectively. The Manhattan plot and Quantile-Quantile plot (Q-Q plot) was drawn by QQman [43] and GGplot2 software [44]. The ideal threshold value was set as 1/201,817 SNPs (-log_10_(*p*) = 5.3) for identifying the marker-trait associations. Finally, to ensure the accuracy of significant SNPs associated with traits, we removed the unique SNPs associated with a trait in the range of LD decay, others were considered as valid associated-trait SNPs.

### Discovery of useful allelic variation for fatty acid composition

The epistatic effect of linked candidate SNPs was evaluated using the epistatic association mapping (EAM) method [45]. When the effect value of SNP is positive, it was taken as increasing effect allele for trait value, conversely, when the effect value is negative as a decreasing effect allele.

SNPs with positive allelic effect values highly associated with oleic acid content and linoleic acid content were analysed, and the SNPs with negative allelic effect value related to erucic acid content and linolenic acid content were counted. Furthermore, the number of varieties with favourable alleles for fatty acid composition were counted.

### Prediction of candidate genes for four fatty acid composition

Candidate genes located within the 100 Kb region upstream or downstream of significant associated-trait SNPs were identified based on GO terms (fatty acid biosynthetic process; very long-chain fatty acid metabolic process; fatty acid elongation; fatty acid metabolic; acetyl-CoA metabolic process; fatty-acyl-CoA reductase activity; phosphatidylinositol transporter activity etc.) for fatty acid synthesis, desaturation, elongation and metabolism. Then the identified candidate genes related to fatty acid composition were further confirmed by BLASTX searching against the *Arabidopsis* protein database.

### Candidate gene expression analysis by qRT-PCR

Seven rape varieties from this studied population with significantly different fatty acid contents were selected to carry out the expression analysis of candidate genes (S2 Table), the total RNA from their frozen root, stem, leaf, flower, and seed were extracted using Eastep^®^ Super total RNA extraction Kit (Promega, Beijing, China) according to the manufacturer’s instructions. RNA quality and quantity were checked using NanoDrop 2000 spectrophotometer (Thermo Fisher Scientific, Wilmington, DE) and RNA integrity was determined by agarose gel electrophoresis.

Three candidate genes *(ACP5*, *FAD2*, *KCS17*) that are significant for fatty acid metabolism were selected to validate the GWAS results by qPCR. The primers for PCR amplification of the three candidate genes were designed with Primer3Plus [46] under strict standards and are provided in supplement file (S3 Table).

A total of 1 μg of total RNA was reverse-transcribed to complementary DNA (cDNA) using the PrimeScript^TM^ RT reagent Kit with gDNA Eraser (Perfect Real Time) (TaKaRa, Japan). according to the manufacturer’s instructions. A 20 ul reaction was prepared with 10 ul of SYBR Green Master mix for real time quantitative PCR (Takara, Japan), 1 ul of each primer pair and 1 ul of cDNA templates, 7 ul ddH_2_O was added to the final reaction volume of 20 ul. The PCR amplification of the target genes was performed on a CFX96 Real-Time PCR system (Biorad, Hercules, CA) with the program as follows: 1 cycle of 95°C for 20s; and 40 cycles of 95°C for 15s, 60°C for 30s and 72°C for 30s; a final melt curve analysis in which the temperature was increased from 55°C to 95°C at a rate of 0.5°C/5s; and a maintenance at 4°C. In every sample, *β-actin* was taken as the house-keeping gene, at least 3 technical replicates were performed for each experiment. The relative quantification of gene expression was calculated using the 2^-ΔΔCT^ method.

## Results

### Phenotypic variation and correlation analysis for four fatty acid compositions in 300 rapeseed accessions

**Fig 1:**
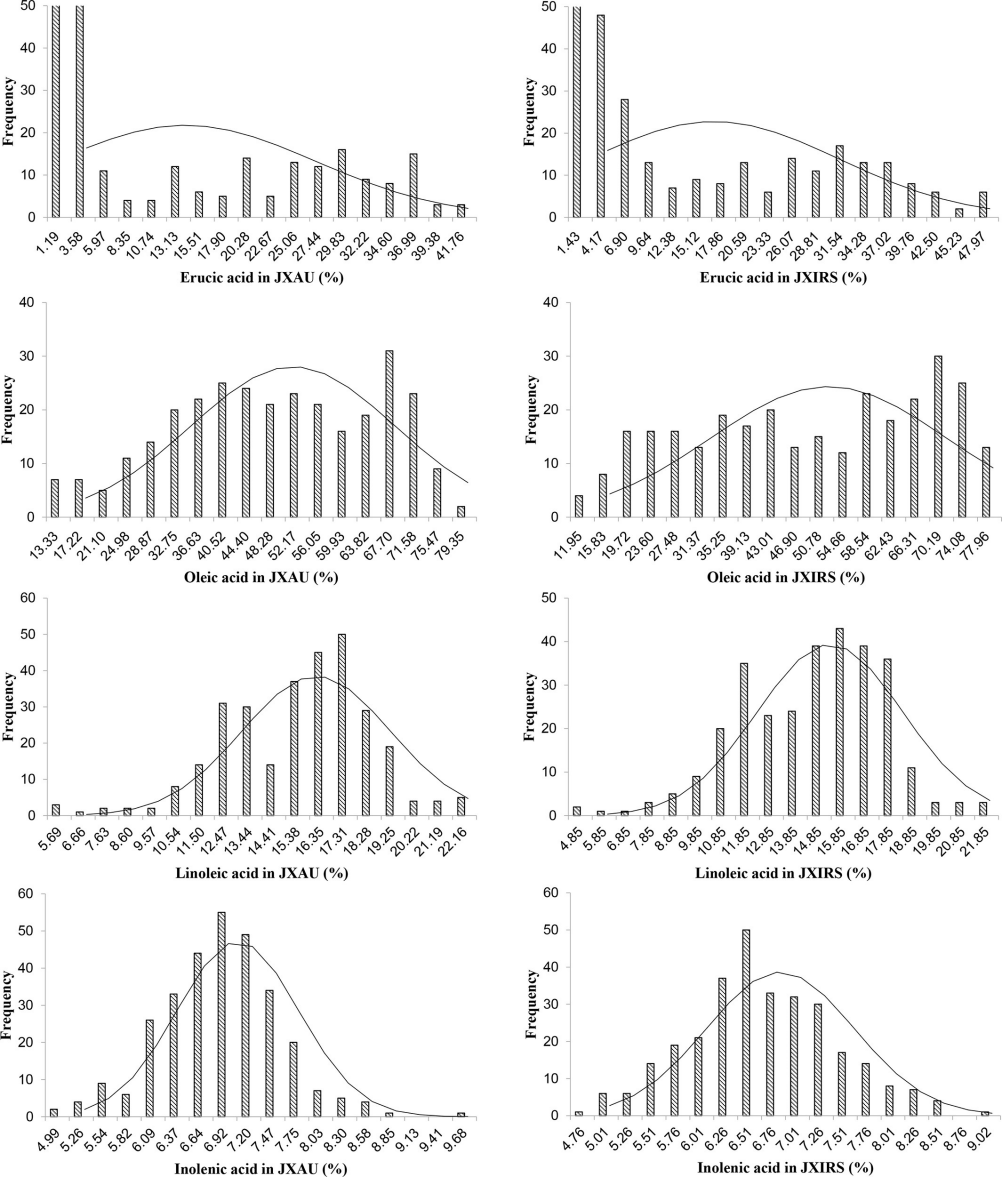
Frequency distribution of four fatty acid content traits of *B*. *napus*.

Continuous and extensive phenotypic variations for each of the four fatty acid composition traits were observed in both environments (JXAU and JXIRS). Linolenic acid content was normally distributed, whilst the contents of erucic acid, oleic acid and linoleic acid had multimodal distributions across the 300 accessions (Fig 1). Descriptive statistical analysis was summarized in Table 1 for oleic acid [C18:1], linoleic acid [C18:2], linolenic acid [C18:3] and erucic acid [C22:1] of 300 accessions under two JXAU and JXIRS environments. The average erucic acid content was 22.23% and 22.72%, ranging from 0 to 56.38% with each CV of 78.92% and 81.90% in JXAU and JXRIS respectively, which was the largest variation of all results; the average content of oleic acid was 48.97% (JXAU) and 49.37% (JXIRS), ranging from10.01% from 81.29% severally; the average linoleic acid content was 15.51% (JXAU) and 14.66% (JXIRS), ranging from 4.35% to 22.64% with the CV of 19.17% and 20.47%, and finally the average content of linolenic acid was 6.89% and 6.67% in two places, ranging from 4.63% to 9.82% with low CV of 9.97% and 11.48%. These data indicated a broad phenotypic variability in four fatty acid compositions within the studied rapeseed population.

**Table 1:** Statistical analysis of four fatty acids of rapeseed.

Trait	Environment	Mean±SE (%)	Range (%)	Coefficient of variation CV (%)	Shapiro wilk
Erucic acid	JXAU	22.23±1.01	0.03–54.71	78.92	W = 0.887946 P = 0.000000
	JXIRS	22.72±1.07	0–56.38	81.90	W = 0.876088 P = 0.000000
Oleic acid	JXAU	48.97±0.95	11.39–81.29	33.63	W = 0.972434 P = 0.000016
	JXIRS	49.37±1.10	10.01–79.90	38.48	W = 0.945312 P = 0.000000
Linoleic acid	JXAU	15.51±0.17	5.21–22.64	19.17	W = 0.91188 P = 0.000001
	JXIRS	14.66±0.17	4.35–22.35	20.47	W = 0.979837 P = 0.000311
Linolenic acid	JXAU	6.89±0.04	4.85–9.82	9.9 7	W = 0.988571 P = 0.018354
	JXIRS	6.67±0.04	4.63–9.14	11.48	W = 0.996549 P = 0.764799

There was strong evidence based on ANOVA that traits varied significantly across the 300 genotypes, and had very significant difference under the interaction between genotype and environment (G×E) (*P*<0.01; Table 2). However, except for oleic acid content, there were no significant effects in other three fatty acids between the two environments. For the correlations between four traits, oleic acid content in both environments had a highly significant negative correlation with erucic acid and linolenic acid content (Table 3). Here, their phenotypic correlation coefficients of -0.8376** and -0.5862** in JXAU and -0.7942** and -0.2775** in JXIRS respectively (*P*<0.01) were observed, but which has a significant positive correlation with linoleic acid content with phenotypic correlation coefficients of 0.4215** in JXAU and 0.5824** in JXIRS. Moreover, linoleic acid content had a highly significant positive correlation with linolenic acid content, with phenotypic correlation coefficients of 0.6879** (JXAU) and 0.5748** (JXIRS).

**Table 2:** Variance analysis of seed fatty acid composition of rapeseed in two environments.

Source of variation	DF	Erucic acid (%)	Oleic acid (%)	Linoleic acid (%)	Linolenic acid (%)
Block	1	39.6692	68.8289	70.1079**	14.5636**
Environment (E)	1	25.0014	123.3815*	1.6539	0.863
Genotype (G)	299	332.3849**	281.0727**	13.891**	1.1998**
G×E	299	286.9632**	274.9371**	11.7758**	0.9602**
Error	599	48.36	31.9287	2.2298	0.3323

Note

*,** present significant at 5% and 1% probability levels respectively.

**Table 3:** The correlation analyses four fatty acid composition of *B*.*napus* in JXAU/JXIRS.

Correlation coefficient	Erucic acid (%)	Oleic acid (%)	Linoleic acid (%)	Linolenic acid (%)
Erucic acid (%)	1	-0.8376**/-0.7942**	-0.6653**/-0.6449**	0.0218**/0.1321
Oleic acid (%)		1	0.4215**/0.5824**	-0.5862**/-0.2775**
Linoleic acid (%)			1	0.6879**/0.5748**
Linolenic acid (%)				1

Note

*,** present significant at 5% and 1% probability levels respectively.

### Genome-wide association analysis for the four fatty acid content traits in the 300 rapeseed accessions

**Fig 2:**
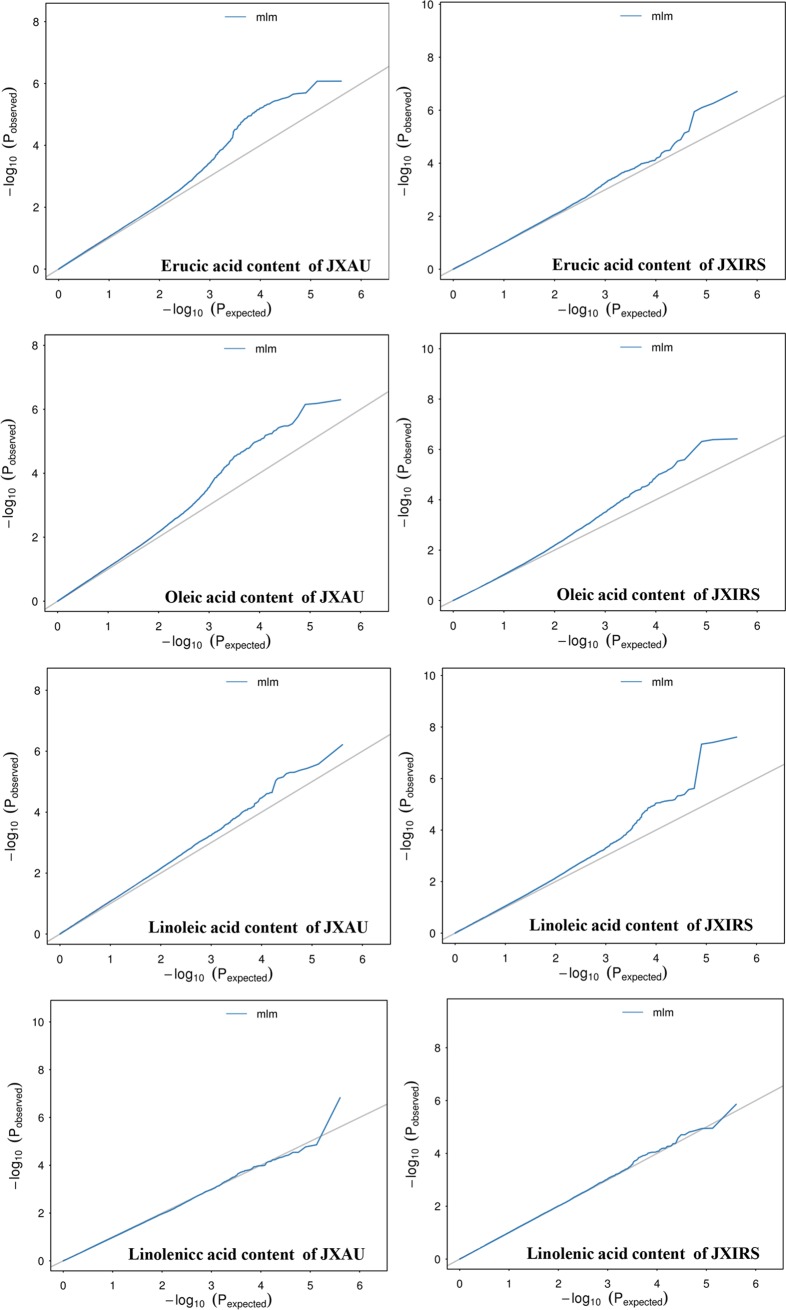
Quantile-quantile plots of estimated-lg (P) from association analysis of four fatty acid content traits using MLM model in two environments (JXAU and JXIRS).

**Fig 3:**
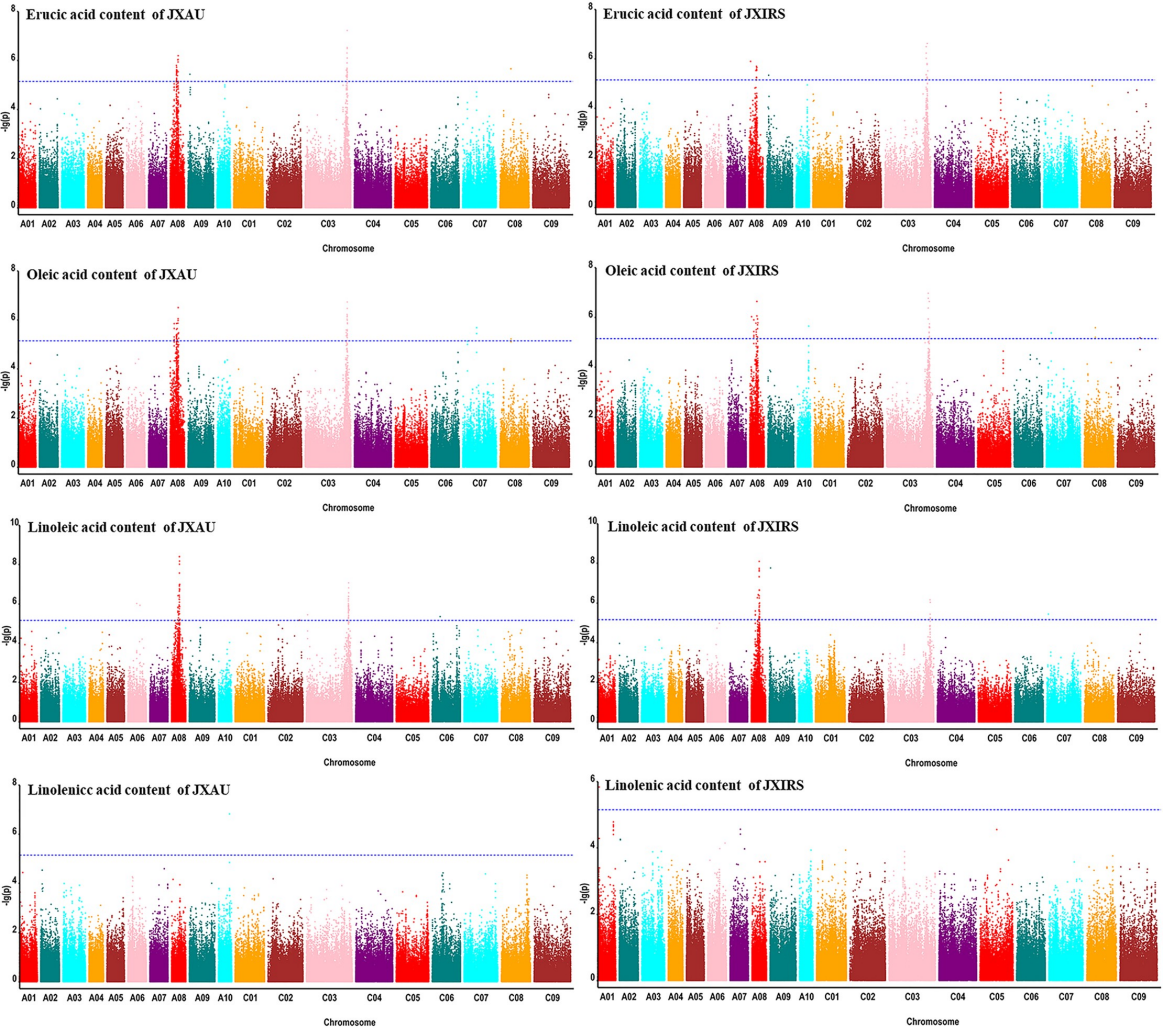
Manhattan of GWAS for four fatty acid content traits of *B*. *napus* by MLM in two environments (JXAU and JXIRS).

To reveal the genetic variations of four fatty acid compositions in *B*. *napus*, GWAS for these traits based on MLM models was conducted, The predictive QQ plots show expected distribution agrees of p-values have a high consistency with the observations (Fig 2), and the significantly associated SNPs per traits were displayed on Manhattan plots (Fig 3). The total results of the significant SNP loci associated with the four fatty acids combined under two environments are given in Table 4. GWAS identified 148 SNPs significantly associated with four fatty acid content traits on 8 chromosomes. However, the majority of trait-linked SNPs were mainly distributed on A08 and C03 chromosomes, with 82 and 48 significant SNP on chromosome A08 and C03, respectively (S4–S7 Tables), and all trait-linked closely SNPs explained 6.09% ~16.55% of observed phenotypic variation.

**Table 4:** Summary of SNP loci associated with four fatty acid composition in *B*. *napus*.

Trait	Environments	Chromosome	P value	R (%)	No. of SNPs
Erucic acid	JXAU	A08	4.09E-06–6.33E-07	7.06–10.57	24
Erucic acid	JXIRS	A08	2.93E-06–1.79E-07	7.03–9.41	9
Erucic acid	JXAU	A09	3.60E-06	7.24	1
Erucic acid	JXIRS	A09	4.53E-06	7.23	1
Erucic acid	JXAU	C03	4.93E-06–5.92E-08	6.68–9.20	29
Erucic acid	JXIRS	C03	4.41E-06–1.81E-07	6.98–8.82	10
Oleic acid	JXAU	A08	4.88E-06–3.10E-07	7.26–11.35	30
Oleic acid	JXIRS	A08	4.88E-06–2.26E-07	7.40–9.90	18
Oleic acid	JXIRS	A10	2.21E-06	8.78	1
Oleic acid	JXAU	C03	4.90E-06–1.84E-07	7.04–8.92	25
Oleic acid	JXIRS	C03	4.11E-06–1.06E-07	7.25–9.34	15
Oleic acid	JXAU	C07	3.05E-07–2.55E-07	6.09–6.40	2
Oleic acid	JXIRS	C07	4.23E-06–4.11E-07	6.15–6.19	2
Oleic acid	JXIRS	C08	2.57E-06	10.02	1
Linoleic acid	JXAU	A06	1.16E-06–9.42E-07	8.76–15.15	2
Linoleic acid	JXAU	A08	4.45E-06–3.883E-9	7.89–16.55	41
Linoleic acid	JXIRS	A08	4.76E-06–7.93E-9	7.48–16.25	32
Linoleic acid	JXIRS	A09	1.70E-08	16.19	1
Linoleic acid	JXAU	A10	3.15E-06	8.36	1
Linoleic acid	JXAU	C03	4.97E-06–8.45E-08	7.59–16.39	36
Linoleic acid	JXIRS	C03	3.62E-06–6.82E-07	7.43–9.64	4
Linoleic acid	JXIRS	C07	3.59E-06	7.54	1
Linolenic acid	JXAU	A10	1.49E-07	12.75	1
Linolenic acid	JXAU	C02	1.75E-06	12.70	1

A total of 78 SNPs of which associated with erucic acid content significantly were detected in GWAS, 12 (15%) were both detected in two environments (S4 Table). In addition, 57 in JXAU and 37 in JXIRS with 22 same SNP loci on 5 chromosomes for oleic acid were detected (S5 Table). Furthermore, we identified 118 SNPs for linoleic acid content trait (80 in JXAU and 38 in JXIRS with 21 uniform SNP loci (S6 Table), but only 2 significant SNP loci in JXAU were found (S7 Table). In addition, 51 peak SNPs (30 of erucic acid, 34 of oleic acid, 37 of linoleic acid and 12 of linolenic acid) were detected on 8 chromosomes (Table 5).

**Table 5:** Peak SNPs associated with four fatty acid contents in seed of *B*. *napus*.

Chromosome	Position	P value	*R*^*2*^	Erucic acid	Oleic acid	Linoleic acid	Linolenic acid
A06	11711750	5.47E-08	0.13304			√	
A08	5233235	2.72E-07	0.04969	√	√	√	
A08	7774253	3.60E-06	0.04354		√		
A08	8025760	3.91E-07	0.06056	√	√		
A08	8268443	3.02E-07	0.06263	√	√		
A08	8347808	4.08E-07	0.05391	√	√		
A08	8390189	4.08E-08	0.09987	√	√	√	
A08	8395297	1.73E-08	0.07549	√	√	√	
A08	8571699	1.70E-07	0.05227	√	√	√	
A08	9171259	2.51E-06	0.04949		√		
A08	9312382	1.75E-07	0.06197		√	√	
A08	9639695	3.88E-07	0.09779	√	√	√	
A08	10146770	2.02E-09	0.09783	√	√	√	
A08	10180037	5.94E-08	0.09362			√	
A08	10233049	9.15E-07	0.07426	√	√	√	
A08	10337576	8.26E-08	0.07682	√	√	√	
A08	10406725	2.62E-08	0.11265			√	
A08	10433764	2.58E-10	0.09296	√	√	√	
A08	10442011	2.48E-08	0.10185	√	√	√	
A08	10461292	1.25E-07	0.09281	√	√	√	
A08	10471805	1.30E-11	0.14511		√	√	
A08	10472012	1.03E-11	0.13556		√	√	
A08	10481532	2.58E-10	0.12307			√	
A08	10495971	1.12E-10	0.10555			√	
A08	10515263	2.19E-11	0.10883	√	√	√	
A08	10582811	9.13E-12	0.11441	√	√	√	
A08	10889000	3.95E-08	0.09829			√	
A08	10958311	4.07E-09	0.093			√	
A08	11136986	4.91E-08	0.06772			√	
A08	11158551	9.94E-08	0.05835	√	√	√	
A09	1752479	2.05E-10	0.13748			√	
A09	2539185	3.50E-07	0.05646	√			
A10	14591650	3.37E-09	0.13573				√
C02	30041401	1.19E-07	0.13582				√
C03	54305474	2.39E-09	0.06921	√	√		
C03	54320531	6.05E-09	0.068	√	√		
C03	55442122	1.24E-07	0.07011	√	√	√	
C03	55522999	7.05E-08	0.073	√	√	√	
C03	55566645	2.19E-08	0.07326	√	√		
C03	55697602	5.89E-09	0.06611	√	√	√	
C03	55712102	5.81E-07	0.06493	√	√	√	
C03	55738483	4.18E-10	0.06887	√	√	√	
C03	55741015	3.81E-08	0.0739	√	√	√	
C03	55851909	1.48E-06	0.06278			√	
C03	55917656	7.45E-08	0.13888			√	
C03	55936314	7.56E-08	0.08354	√	√	√	
C03	56690589	6.61E-08	0.09359			√	
C07	6271446	3.74E-07	0.04589			√	
C07	18403416	4.73E-07	0.04454		√		
C08	4845350	2.88E-07	0.0493	√			
C08	14472595	3.58E-08	0.08004	√	√		

Note

"√" indicates the corresponding trait that the significant associated SNP locus.

For the SNP locus analysis, there were some SNP loci significantly associated with several traits simultaneously. For example, a total of 30 SNP loci on A08, C03 and C06 chromosomes were detected in three traits of erucic acid content, oleic acid content and linoleic acid content. Apart from what is outlined above, 16 SNP loci linked to erucic acid content and oleic acid content, 25 loci for oleic acid content and linoleic acid content, and 7 SNPs for erucic acid content and linoleic acid content concurrently. These consistent SNPs detected simultaneously in different traits indicated that they might control the different fatty acid composition traits synchronously. What’s more, we found two remarkable associated regions on chromosome A08 and C03 were consistent with the QTL results based on bi-parental mapping previously [19, 31, 47].

To identify highly favourable alleles with for fatty acid composition in *B*. *napus*, we investigated the allelic effects of all the significantly associated SNPs. We observed 108 highly favourable alleles for increasing oleic acid and linoleic acid content, also for decreasing erucic acid content simultaneously (S8 Table). Specially, we found 66 highly favourable alleles for reducing the content of erucic acid and linolenic acid (S9 Table), 82 and 105 highly favourable alleles contributed to increase oleic acid and linoleic acid content respectively (S10 Table, S11 Table). Discovery of SNP loci and those favourable alleles in current study will provide insight into the genetic basis of four fatty acid biosynthesis in rapeseed.

### Candidate genes identification of four fatty acids in *B*. *napus*

To further reveal the molecular function of the SNPs significantly associated with the four traits, we extracted the genes within the 100 Kb upstream or downstream regions of the trait-associated SNPs in the reference genome of *B*. *napus* “Darmor v4.1”. We found 802 genes were identified located in the candidate regions around 52 SNPs (S12 Table), 29 of these candidate genes were enriched into fatty acid biosynthetic process, fatty acid metabolic process and Lipid transport and metabolism in the GO terms annotation analysis (S13 Table). 20 fatty acid composition candidate genes were homologous to *A*. *thaliana* genes involved in metabolic networks of fatty acids. These candidate genes were located in four chromosomes (A08, A09, A10 and C03), 16 of which were distributed on the A subgenome, the chromosome A08 have the most genes (12), and chromosome A09 and A10 have 2 candidate genes, respectively, chromosome C03 have other 4 fatty acid candidate genes (Table 6), which suggested the A subgenome pay a more important role than C subgenome in the metabolic networks of fatty acids of rapeseed.

**Table 6:** Candidate genes tagged by the associated SNPs with fatty acid biosynthesis and metabolism in *B*. *napus* and their orthologs in *A*. *thaliana*.

Gene	Chr.	Gene start	Gene end	SNP location	Distance (Kb)	Arabidopsis genes	LD interval (bp) (*R*^*2*^>0.6)	Alias	Description
BnaA08g08120D	A08	8006912	8010592	8025760	18.848	AT4G20930			6-phosphogluconate dehydrogenase family protein
BnaA08g08850D	A08	8588827	8591995	8571653	17.174	AT4G18550	7205628–8404857	DSEL	Dermatan sulfate epimerase-like
BnaA08g09510D	A08	9087020	9088618	9171259	84.239	At4g20830	8404821–8659578		FAD-binding Berberine family protein
BnaA08g09990D	A08	9372442	9374898	9312398	60.044	AT4G20930	8404821–9237980		6-phosphogluconate dehydrogenase family protein
BnaA08g11130D	A08	10187701	10189221	10146770	40.931	AT4G34520	9296073–9461455	FAE1	Fatty acid elongase 1
BnaA08g11140D	A08	10193789	10195213	10146770	47.019	AT4G34510	10146770–10233049	KCS17	3-ketoacyl-CoA synthase 17
BnaA08g11440D	A08	10385625	10391818	10433764	41.946	AT4G33790	10146770–10233049	CER4	Eceriferum 4
BnaA08g11650D	A08	10512857	10515956	10433764	79.093	AT4G34030	10337911–10495999	MCCB	3-methylcrotonyl-CoA carboxylase
BnaA08g11810D	A08	10599612	10600625	10507743	91.869	AT4G233355	10337911–10495999		Bifunctional inhibitor/lipid-transfer protein/seed storage 2S albumin superfamily protein
BnaA08g11640D	A08	10507282	10508723	10582811	75.529	AT4G34050	10507743–10625538	CCOAOMT1	Caffeoyl-CoA O-methyltransferase 1
BnaA08g12350D	A08	10994361	10997455	10958311	36.05	AT4G31810	10496004–10641083		Enoyl-CoA hydratase/isomerase family protein
BnaA08g12370D	A08	11043381	11046227	10958311	85.07	AT4G31750	10958311–11136986	WIN2	HOPW1-1-interacting 2 (WIN2)
BnaA09g03610D	A09	1823513	1824394	1752479	71.034	AT5G27200	10958311–11136986	ACP5	Acyl carrier protein 5
BnaA09g05410D	A09	2642292	2646291	2554018	88.274	AT5G23260	1733014–1984004	TT16	Transparent testa 16
BnaA10g20970D	A10	14540117	14540680	14591650	50.970	AT1G77590	2539185–2943582	LACS9	Long chain acyl-CoA synthetase 9
BnaA10g21150D	A10	14626208	14627791	14591650	34.558	AT5G67030	14516759–14778935	ZEP	Zeaxanthin epoxidase
BnaC03g03500D	C03	1710052	1711864	1664875	45.177	AT3G12120	14516759–14778935	FAD2	Fatty desaturase 2
BnaC03g65730D	C03	55426564	55428053	55442089	15.525	AT5G13930	1557947–1756319	ATCHS	Chalcone synthase
BnaC03g65980D	C03	55684352	55685779	55618985	65.367	AT4G34520	54559600–55522974	FAE1	Fatty acid elongase 1
BnaC03g66040D	C03	55810262	55811686	55712102	98.160	AT4G34510	55256311–55847797	KCS17	3-ketoacyl-CoA synthase 17

Some genes tagged by associated SNPs in our study represent known fatty acid composition genes, and were enriched in biological processes of FAs biosynthesis. For erucic acid, two *Fatty acid elongation 1* (*FAE1*) orthologous genes (*BnaA08g11130D* and *BnaC03g65980D*) were found in the distance of 40.93 Kb from SNP *Bn-A08-10146770* and 65.37 Kb form *Bn-C03-55618985* respectively, which were well-known *Bna*.*FAE1* homologs controlling erucic acid content found in previous studies [25, 31, 48–50]. Moreover, another two candidate genes of *BnaA08g11140D* and *BnaC03g66040D* orthologous to *3-ketoacyl-CoA synthase 17* (*KSC17*) in the distance of 47.02 Kb from SNP *Bn-A08-10146770* and 98.16 Kb form *Bn-C03- 55712102* were detected respectively, which is also a member of the 3-ketoacyl-CoA synthase family involved in the biosynthesis of very long chain fatty acids [51]. For the synthesis of unsaturated fatty acids, which are affected by the fatty acid desaturase activity in plant, the fatty acid desaturase genes have been proved as the major genes for the control of oleic acid content as same as the ratio of polyunsaturated fatty acid, we found the important candidate genes of *BnaC03g03500D* orthologous to *Fatty acid desaturase 2* (*FAD2*) in the distance of 45.18 from *Bn-C03-1664875*, which is the major enzyme responsible for the synthesis of 18:2 fatty acids in plant endoplasmic reticulum [3], and *FAD2* gene had been mapped in *B*. *napus* on chromosomes A1, A5, C1, and C5 in previous studies [4, 52]. Furthermore, many other important candidate genes for fatty acid biosynthesis were detected, which were orthologous to *A*. *thaliana*, such as *Dermatan sulfate epimerase-like* (*DSEL*), *Acyl carrier protein 5* (*ACP5*), *Long chain acyl-CoA synthetase 9* (*LACS9*) and so on, these candidate genes except for *BnaA*.*FAE1* (*BnaA08g11130D*) and *BnaC*. *FAE1* (B*naC03g65980D*) have not been verified in previous genetic analyses, which are likely play important roles in fatty acid synthesis and transport. Therefore, these candidate genes detected in this study should be certified by further analysis in the future.

### The expression of three candidate genes in diverse rapeseed accessions and tissues

In order to validate the candidate genes significantly associated fat acids compositions, we selected three key genes involved in fat acid synthesis and measured their gene expression in five different tissues (root, stem, leaf, flower and seed) of seven diverse rapeseed accessions using qRT-PCR. We found the three candidate genes have high expression in seeds or flowers of these selected rapeseed (S1 Fig), and the expression of *ACP5* have a strong positive correlation with erucic acid (0.68), have strong negative correlation with oleic acid (-0.67) and linoleic acid (-0.71), suggested the high expression of *ACP5* could promote the erucic acid synthesis, but decrease the oleic acid and linoleic acid. *FAD2* and *KCS17* have positive correlation with oleic acid and linoleic acid, indicated they could increase the two fat acids in the studied population (Table 7).

**Table 7:** The correlation analyses between the expression of three candidate genes and four fatty acid composition of *B*.*napus* in JXAU.

Correlation coefficient (*R*)	*ACP5*	*FAD2*	*KCS17*
**Erucic acid**	0.68	-0.15	-0.46
**Oleic acid**	-0.67	0.29	0.50
**Linoleic acid**	-0.71	0.23	0.51
**Linolenic acid**	-0.39	0.16	-0.22

## Discussion

Fatty acid compositions in seed of rapeseed play important roles in improving the edible oil nutritional and storage quality. In this study, we investigated the four important fatty acid content traits of erucic, oleic, linoleic and linolenic for 300 rapeseed accessions, the four traits exhibited large variation in two different environments, and there proved to be have the weak or no genetic relationship among 300 accessions in our previous study [35,36], validating the suitability of GWAS for these traits in this study population [53]. In addition, there are high correlations among these fatty acid traits, significant negative correlation between the content of erucic acid and oleic acid was identified, and linoleic acid content trait had high positive correlation with other three traits, they were co-localized in a small region on chromosome A08 and C03 chromosomes, the strong correlation between these traits was also observed in previous studies [19, 21, 45, 54]. Furthermore, in current study, although the materials were grown in two near environments with near locations, but there were very distinct differences in these fatty acid compositions under the interaction between genotype and environment, we speculate the acid soil with low pH value (4.67) in JXIRS was the main reason resulting in very distinct differences of four fatty acids content [55].

Fatty acid composition are typical quantitative traits, based on the genetic markers (SNP, SSR, RFLP and AFLP), bi-parent population and statistical methods, many QTLs for fatty acid composition distributed on most of chromosomes of *B*. *napus* were detected in the past few decades [3, 4, 19–21, 25, 30]. Recent efforts have also been made in detecting the genetic loci responsible for fatty acid composition in *B*. *napus* based on the Illumina Infinium *Brassica* 60K SNP array, some genome wide analysis were carried out to identified genomic region and candidate genes associated with fatty acid content in the past few years [26, 27, 31]. In this study, using 201,187 genome-wide SNP markers developed by SLAF-seq technology [35,36], we carried out the GWAS for four fatty acid traits in 300 rapeseed inbred lines, and hundreds of SNP loci highly associated with the four fatty acid compositions traits were identified on 8 chromosomes of *B*. *napus*, many of these SNPs were simultaneously detected in two environments, most of significantly associated SNPs (more than 80%) for the content of erucic acid, oleic acid and linoleic acid were identified on A08 and C03 chromosomes, which was in accordance with above QTL and GWAS studies for fatty acid composition, from this we can infer that the genomic region controlling the fatty acid biosynthesis were mainly distributed on A08 and C03 chromosomes. In addition, we also found a few trait-associated SNPs on other chromosomes, some identical chromosome regions were exist in Qu et al. (2017) [31], for example, the significant SNPs associated with olenic acid on A09 chromosomes and the candidate gene of *BnaA09g05410D* (orthologous to *Transparent testa 16 TT16*) was identified both in two studies, but other related gene groups found in A02 and C01 linkages of Qu et al. (2017) were not detected in current study[31], which probably attribute to the different population used in different studies. Under the reference-guided analysis, As the physical position and alleles of SNP markers used in this study are known, therefore, breeders and researchers could easily obtain valuable information for other related rapeseed research based on our results. In addition, by evaluating the allelic effects of trait-associated SNPs, we observed many favourable alleles for decreasing the erucic acid content, which had positive effects for increasing the oleic and linoleic acid content in the same time, these SNPs could be used for low-erucic and high-oleic acid rapeseed breeding in the future.

The genetic basis of seed fatty acid biosynthesis and modification pathways have been well characterised in *Arabidopsis thaliana* [9]. Well established desaturation and elongation pathways, along with substrate: product relationships assigned to specific enzymes [10], which was a co-operation of many genes of seed development, energy metabolism, fatty acid and triacylglycerol (TAG) biosynthesis pathways [9, 56]. *De novo* synthesis of fatty acids occur in the seed plastid, where the palmtic acid (16:0), stearic acid (18:0) and oleic acid (18:1) are formed and then released to the endoplasmic reticulum (ER) by two kinds of acyl-ACP thioesterase enzymes (*FATA* and *FATB* with high affinity to 18:1-ACP and 16:0-ACP respectively) [57, 58]. These fatty acids were modified by desaturation enzymes (*FAD2* and *FAD3*) to produce the polyunsaturated linoleic (18:2) and linolenic acid (18:3) [52, 59], the gene expression analysis in this study supported further that the *FAD2* gene would promote the linolenic acid synthesis. In addition, the fatty acids could be elongated to erucic acid by *FAE1* gene or esterified to glycerol to produce the TAG stored as a major seed oil form in plants [60]. In current study, a total of 14 candidate genes were detected on A08 chromosomes, including *DSEL*, *FAE1*, *MCCB*, *CCOAOMT1* and *WIN2* etc. In addition, there were 4 fatty acid biosynthesis genes were found on C03 chromosomes, including *FAD2*, *ATCHS*, *FAE1* and *KCS17* etc., Specially, two known homoeologous genes (namely *BnaA*.*FAE1* and *BnaC*.*FAE1*) controlling erucic acid content were identified in our study [25, 31, 50, 61], and candidate genes *BnaA08g11140D* and *BnaC03g66040D* orthologous to *KCS17* of *A*. *thaliana* involving in the biosynthesis of very long chain fatty acids were also found on chromosomes A8 and C3. Moreover, the gene of *BnaA08g11440D* near the SNP locus of *Bn-A08-10317341* (68.284 Kb) is orthologous to *A*. *thaliana* CER4 encoding an Alcohol-Forming Fatty Acyl-Coenzyme A Reductase involved in the synthesis of very long chain fatty acids [62]. These results obviously indicate that there are genomic regions controlling the seed fatty acid biosynthesis on A08 and C03 chromosomes. By the way, some important fatty acid candidate genes were also scanned on other chromosomes. On A09 chromosome, the candidate gene of *BnaA09g03610D* orthologous to *Acyl carrier protein 5* (*ACP5*) was located in the distance of 71.03 Kb from SNP *Bn-A09-1752479* on chromosome A09, which is a small acidic proteins functioning as important cofactors in the *de novo* synthesis of fatty acids, overexpression of *AtACP5* further led to an a decrease of oleic acid (C18:1) and an increase of palmitic acid (C16:0) [63], the gene expression of *ACP5* in this study have strong negative relationship with oleic acid, suggested it seem to has the same gene function as *AtACP5*. In addition, On A10 chromosome, three candidate genes were ascribed to fatty acid, *BnaA10g20970D* orthologous to *A*. *thaliana Long chain acyl-CoA synthetase 9* (*LACS9*) encoding major plastid long chain acyl-CoA synthetase with a slight substrate preference of oleic acid over any of the other fatty acids. *Protein-tyrosine phosphatase-like* (*PTPLA*) (*BnaA10g21780D*) with acyl-CoA dehydratase activity has a potential role in synthesis of VLCFAs (very long chain fatty acids) under the interaction with *Eceriferum 10* (*CER10*), which is a component of the microsomal fatty acid elongase complex. On C06 chromosome, *FatA* acyl-ACP thioesterase (*FATA*) affects the oil content and fatty acid composition of the seeds in *Arabidopsis* when reducing expression of *FatA* thioesterases [64]. Our GWAS analysis led to the identification of promising candidate genes for fatty acid biosynthesis and metabolism efficiently.

## Conclusion

In this study, based on GWAS with MLM model analysis, significant association signals for the content of erucic acid, oleic acid, linoleic acid and linolenic acid in seeds of *B*. *napus* were found on A06, A08, A09, A10, C02, C03, CO6, C07 and C08 chromosomes in two environments. The genomic regions controlling the fatty acid biosynthesis were inferred to distribute mainly on A08 and C03 chromosomes. 20 orthologs of the functional candidate genes related to fatty acid biosynthesis in a distance of 100 Kb around these significantly SNP-trait associations were identified by BLAST analysis and comparison of previous linkage mappings, including the known vital fatty acid biosynthesis genes of *BnaA*.*FAE1* and *BnaC*. *FAE1* on the A08 and C03 chromosomes, and other potential candidate genes involving in the fatty acid biosynthesis pathway, such as the orthologs genes of *FAD2*, *LACS09*, *KCS17*, *CER4*, *TT16* and *ACBP5*. This study lays a foundation for uncovering the genetic variations and the improvement of fatty acid composition in *B*. *napus*.

## Supporting information

S1 Fig The gene expression of *ACP5* (A), *FAD2* (B), *KCS17* (C) in diverse rapeseed accessions and tissues.
(DOCX)

S1 Table Material information of 300 inbred lines of *B*. *napus*. (XLS)

S2 Table The fatty acid content of selected seven inbred lines of *B*. *napus*. (XLS)

S3 Table Excel file containing the primer sequences used for real-time PCR. (XLSX)

S4 Table SNPs significantly associated with erucic acid content in *B*. *napus* by MLM. (XLSX)

S5 Table SNPs significantly associated with oleic acid content in *B*. *napus* by MLM. (XLSX)

S6 Table SNPs significantly associated with linoleic acid content in *B*. *napus* by MLM. (XLSX)

S7 Table SNPs significantly associated with linolenic acid content in *B*. *napus* by MLM. (XLSX)

S8 Table SNP loci with positive effects for oleic acid, linoleic acid content and negative effects for erucic acid content in *B*. *napus*. (XLSX)

S9 Table SNP loci with negative effects for erucic acid content in *B*. *napus*. (XLSX)

S10 Table SNP loci with positive effects for oleic acid content in *B*. *napus*. (XLSX)

S11 Table SNP loci with positive effects for linoleic acid content in *B*. *napus*. (XLSX)

S12 Table Genes closely linked with trait-associated SNPs in *B*. *napus*. (XLSX)

S13 Table Candidate genes for fatty acid biosythesis and metablism closely linked with SNPs in *B*. *napus* based on GO annotation. (XLSX)

## References

[1] Shahzadi T, Khan FA, Zafar F, Ismail A, Amin E, Riaz S. An Overview of Brassica Species for Crop Improvement. American-Eurasian Journal of Agricultural and Environmental Sciences. 2015;15(8):1568–73. 10.5829/idosi.aejaes.2015.15.8.12746

[2] Carvalho IS, Miranda I, Pereira H. Evaluation of oil composition of some crops suitable for human nutrition. Industrial Crops & Products. 2006;24(1):75–8. 10.1016/j.indcrop.2006.03.005

[3] Hu X, Sullivan-Gilbert M, Gupta M, Thompson SA. Mapping of the loci controlling oleic and linolenic acid contents and development of fad2 and fad3 allele-specific markers in canola (*Brassica napus* L.). Theoretical and Applied Genetics. 2006;113(3):497–507. 10.1007/s00122-006-0315-1 . 16767448

[4] Smooker AM, Wells R, Morgan C, Beaudoin F, Cho K, Fraser F, et al The identification and mapping of candidate genes and QTL involved in the fatty acid desaturation pathway in Brassica napus. Theoretical and Applied Genetics. 2011;122(6):1075–90. 10.1007/s00122-010-1512-5 . 21184048

[5] Shahidi F. Rapeseed and Canola: Global Production and Distribution. Canola and Rapeseed 1990 p. 3–13.

[6] Velasco L, Fernández-Martínez JM. Breeding Oilseed Crops for Improved Oil Quality. Journal of Crop Production. 2002;5(1–2):309–44. 10.1300/J144v05n01_13

[7] Farmer EE, Weber H, Vollenweider S. Fatty acid signaling in Arabidopsis. Planta. 1998;206(2):167–74. 10.1007/s004250050388 . 9736997

[8] Thelen JJ, Ohlrogge JB. Metabolic Engineering of Fatty Acid Biosynthesis in Plants. Metabolic Engineering. 2002;4(1):12–21. 10.1006/mben.2001.0204 . 11800570

[9] Bates PD, Stymne S, Ohlrogge J. Biochemical pathways in seed oil synthesis. Current Opinion in Plant Biology. 2013;16(3):358–64. 10.1016/j.pbi.2013.02.015 . 23529069

[10] Barker GC, King GJ. Novel insights into seed fatty acid synthesis and modification pathways from genetic diversity and quantitative trait Loci analysis of the *Brassica* C genome. Plant Physiology. 2007;144(4):1827–42. 10.1104/pp.107.096172 . 17573542 PMC1949901

[11] Qiu D, Morgan CJ, Long Y, Liu J, Li R, Zhuang X, et al A comparative linkage map of oilseed rape and its use for QTL analysis of seed oil and erucic acid content. Theoretical and Applied Genetics. 2006;114(1):67–80. 10.1007/s00122-006-0411-2 . 17033785

[12] Gang C, Geng J, Rahman M, Liu X, Tu J, Fu T, et al Identification of QTL for oil content, seed yield, and flowering time in oilseed rape (*Brassica napus*). Euphytica. 2010;175(2):161–74. 10.1007/s10681-010-0144-9

[13] Jiang C, Shi J, Li R, Long Y, Wang H, Li D, et al Quantitative trait loci that control the oil content variation of rapeseed (*Brassica napus* L.). Theoretical and Applied Genetics. 2014;127(4):957 10.1007/s00122-014-2271-5 . 24504552

[14] Zhao J, Becker HC, Zhang D, Zhang Y, Ecke W. Oil Content in a European × Chinese Rapeseed Population. Crop Science. 2005;45(1):51–9. 10.2135/cropsci2005.0051

[15] Jianyi Z, Becker HC, Dongqing Z, Yaofeng Z, Wolfgang E. Conditional QTL mapping of oil content in rapeseed with respect to protein content and traits related to plant development and grain yield. Theoretical and Applied Genetics. 2006;113(1):33–8. 10.1007/s00122-006-0267-5 . 16614833

[16] Mahmood T, Rahman MH, Stringam GR, Yeh F, Good AG. Identification of quantitative trait loci (QTL) for oil and protein contents and their relationships with other seed quality traits in Brassica juncea. Theoretical and Applied Genetics. 2006;113(7):1211–20. 10.1007/s00122-006-0376-1 . 16960718

[17] Uzunova M, Weissleder K, Robbelen GEW. Mapping the genome of rapeseed (*Brassica napus* L.). I. Construction of an RFLP linkage map and localization of QTLs for seed glucosinolate content. Theoretical and Applied Genetics. 1995;90(2):194–204. 10.1007/BF00222202 . 24173891

[18] Hasan M, Friedt W, Pons-Kühnemann J, Freitag NM, Link K, Snowdon RJ. Association of gene-linked SSR markers to seed glucosinolate content in oilseed rape (Brassica napus ssp. napus). Theoretical and Applied Genetics. 2008;116(8):1035–49. 10.1007/s00122-008-0733-3 . 18322671

[19] Zhao J, Dimov Z, Becker HC, Ecke W, Möllers C. Mapping QTL controlling fatty acid composition in a doubled haploid rapeseed population segregating for oil content. Molecular Breeding. 2008;21(1):115–25. 10.1007/s11032-007-9113-y

[20] Wen J, Xu J, Yan L, Xu H, Wu J, Meng J, et al Mapping QTLs Controlling Beneficial Fatty Acids Based on the Embryo and Maternal Plant Genomes in *Brassica napus* L. Journal of the American Oil Chemists Society. 2015;92(4):541–52. 10.1007/s11746-015-2618-3

[21] Burns MJ, Barnes SR, Bowman JG, Clarke MH, Werner CP, Kearsey MJ. QTL analysis of an intervarietal set of substitution lines in *Brassica napus*: (i) Seed oil content and fatty acid composition. Heredity. 2003;90(1):39–48. Epub 2003/01/11. 10.1038/sj.hdy.6800176 . 12522424

[22] Scheffler JA, Sharpe AG, Schmidt H, Sperling P, Parkin IAP, Lühs W, et al Desaturase multigene families of *Brassica napus* arose through genome duplication. Theoretical and Applied Genetics. 1997;94(5):583–91. 10.1007/s001220050454

[23] Schierholt A, Becker HC, Ecke W. Mapping a high oleic acid mutation in winter oilseed rape (Brassica napus L.). Theoretical and Applied Genetics. 2000;101(5–6):897–901. 10.1007/s001220051559

[24] David E, Jacqueline B, Snowdon RJ. Accessing complex crop genomes with next-generation sequencing. Theoretical and Applied Genetics. 2013;126(1):1–11. 10.1007/s00122-012-1964-x . 22948437

[25] Li F, Chen B, Xu K, Wu J, Song W, Bancroft I, et al Genome-wide association study dissects the genetic architecture of seed weight and seed quality in rapeseed (Brassica napus L.). DNA Research,21,4(2014-2-7). 2014;21(4):355–67. 10.1093/dnares/dsu002 . 24510440 PMC4131830

[26] Gajardo HA, Wittkop B, Sotocerda B, Higgins EE, Parkin IAP, Snowdon RJ, et al Association mapping of seed quality traits in Brassica napus L. using GWAS and candidate QTL approaches. Molecular Breeding. 2015;35(6):143 10.1007/s11032-015-0340-3

[27] Körber N, Bus A, Li J, Parkin IA, Wittkop B, Snowdon RJ, et al Agronomic and Seed Quality Traits Dissected by Genome-Wide Association Mapping in Brassica napus. Frontiers in Plant Science. 2016;7(7):386 10.3389/fpls.2016.00386 . 27066036 PMC4814720

[28] Liu S, Fan C, Li J, Cai G, Yang Q, Wu J, et al A genome-wide association study reveals novel elite allelic variations in seed oil content of Brassica napus. Theoretical and Applied Genetics. 2016;129(6):1203–15. 10.1007/s00122-016-2697-z . 26912143

[29] Raman H. Genome-wide association analyses reveal complex genetic architecture underlying natural variation for flowering time in canola. Plant Cell and Environment. 2016;39(6):1228–39. 10.1111/pce.12644 . 26428711

[30] Wang N, Chen B, Xu K, Gao G, Feng L, Qiao J, et al Association mapping of flowering time QTLs and insight into their contributions to rapeseed growth habits. Frontiers in Plant Science. 2016;7 10.3389/fpls.2016.00338 . 27047517 PMC4805649

[31] Qu C, Jia L, Fu F, Zhao H, Lu K, Wei L, et al Genome-wide association mapping and Identification of candidate genes for fatty acid composition in *Brassica napus* L. using SNP markers. Bmc Genomics. 2017;18(1):232 10.1186/s12864-017-3607-8 . 28292259 PMC5351109

[32] Raman H, Raman R, Kilian A, Detering F, Carling J, Coombes N, et al Genome-wide delineation of natural variation for pod shatter resistance in *Brassica napus*. PLoS One. 2014;9(7):e101673 Epub 2014/07/10. 10.1371/journal.pone.0101673 25006804 PMC4090071

[33] Li L, Long Y, Zhang L, Dalton-Morgan J, Batley J, Yu L, et al Genome wide analysis of flowering time trait in multiple environments via high-throughput genotyping technique in *Brassica napus* L. PLoS One. 2015;10(3):e0119425 Epub 2015/03/20. 10.1371/journal.pone.0119425 25790019 PMC4366152

[34] Davey JW, Hohenlohe PA, Etter PD, Boone JQ, Catchen JM, Blaxter ML. Genome-wide genetic marker discovery and genotyping using next-generation sequencing. Nature reviews Genetics. 2011;12(7):499–510. Epub 2011/06/18. 10.1038/nrg3012 . 21681211

[35] Zhou Q, Zhou C, Zheng W, Mason AS, Fan S, Wu C, et al Genome-Wide SNP Markers Based on SLAF-Seq Uncover Breeding Traces in Rapeseed (*Brassica napus* L.). Frontiers in Plant Science. 2017;8:648 10.3389/fpls.2017.00648 . 28503182 PMC5409215

[36] Zhou Q, Han D, Mason AS, Zhou C, Zheng W, Li Y, et al Earliness traits in rapeseed (Brassica napus): SNP loci and candidate genes identified by genome-wide association analysis. DNA Research, 2017;25(3):229–224. 10.1093/dnares/dsx052 29236947 PMC6014513

[37] Tang QY, Zhang CX. Data Processing System (DPS) software with experimental design, statistical analysis and data mining developed for use in entomological research. Insect Sci. 2013;20(2):254–60. Epub 2013/08/21. 10.1111/j.1744-7917.2012.01519.x . 23955865

[38] Murray MG, Thompson WF. Rapid isolation of high molecular weight plant DNA. Nucleic Acids Research. 1980;8(19):4321–5. 10.1093/nar/8.19.4321 . 7433111 PMC324241

[39] Sun X, Liu D, Zhang X, Li W, Liu H, Hong W, et al SLAF-seq: An Efficient Method of Large-Scale De Novo SNP Discovery and Genotyping Using High-Throughput Sequencing. Plos One. 2013;8(3):e58700 10.1371/journal.pone.0058700 . 23527008 PMC3602454

[40] Alexander DH, Novembre JK. Fast model-based estimation of ancestry in unrelated individuals. Genome Research. 2009;19(9):1655–64. 10.1101/gr.094052.109 . 19648217 PMC2752134

[41] Hardy OJ, Vekemans X. SPAGeDI: A versatile computer program to analyse spatial genetic structure at the individual or population levels. Molecular Ecology Notes. 2002;2(4):618–20.

[42] Bradbury P, Zhang Z, Kroon D, Casstevens TY, Buckler E. TASSEL: software for association mapping of complex traits in diverse samples. Bioinformatics. 2007;23(19):2633–5. 10.1093/bioinformatics/btm308 . 17586829

[43] Turner SD. qqman: an R package for visualizing GWAS results using Q-Q and manhattan plots. Journal of Social Structure. 2018;3(25):731.

[44] Gomezrubio V. ggplot2—Elegant graphics for data analysis (2nd Edition). Journal of Statistical Software. 2017;077(1):1–3.

[45] Hai-Yan L, Xiao-Fen L, Shi-Ping W, Yuan-Ming Z. Epistatic association mapping in homozygous crop cultivars. Plos One. 2011;6(3):e17773 10.1371/journal.pone.0017773 . 21423630 PMC3058038

[46] Untergasser A, Cutcutache I, Koressaar T, Ye J, Faircloth BC, Remm M, et al Primer3—new capabilities and interfaces. Nucleic Acids Reseach. 2012;40(15):e115–e115. 10.1093/nar/gks596 . 22730293 PMC3424584

[47] Xu JF, Long Y, Wu JG, Xu HM, Zhao ZG, Wen J, et al QTL identification on two genetic systems for rapeseed glucosinolate and erucic acid contents over two seasons. Euphytica. 2015;205(3):647–57. 10.1007/s10681-015-1379-2

[48] Fourmann M, Barret P, Renard M, Pelletier G, Delourme R, Brunel D. The two genes homologous to Arabidopsis FAE1 co-segregate with the two loci governing erucic acid content in *Brassica napus*. Theoretical and Applied Genetics. 1998;96(6–7):852–8. 10.1007/s001220050812

[49] Qian W, Meng J, Li M, Frauen M, Sass O, Noack J, et al Introgression of genomic components from Chinese *Brassica rapa* contributes to widening the genetic diversity in rapeseed (*B*. *napus* L.), with emphasis on the evolution of Chinese rapeseed. Theoretical and Applied Genetics. 2006;113(1):49–54. 10.1007/s00122-006-0269-3 . 16604336

[50] Wu G, Wu Y, Xiao L, Li X, Lu C. Zero erucic acid trait of rapeseed (*Brassica napus* L.) results from a deletion of four base pairs in the fatty acid elongase 1 gene. Theoretical and Applied Genetics. 2008;116(4):491–9. 10.1007/s00122-007-0685-z 18075728

[51] Jér?Me J, Sylvain R, Brice B, Christel G, Jeanny LT, Patrick M, et al The VLCFA elongase gene family in *Arabidopsis thaliana*: phylogenetic analysis, 3D modelling and expression profiling. Plant Molecular Biology. 2008;67(5):547 10.1007/s11103-008-9339-z 18465198

[52] Yang Q, Fan C, Guo Z, Qin J, Wu J, Li Q, et al Identification of FAD2 and FAD3 genes in *Brassica napus* genome and development of allele-specific markers for high oleic and low linolenic acid contents. Theoretical and Applied Genetics. 2012;125(4):715–29. 10.1007/s00122-012-1863-1 . 22534790

[53] Huang X, Han B. Natural variations and genome-wide association studies in crop plants. Annual Review of Plant Biology. 2014;65(1):531–51. 10.1146/annurev-arplant-050213-035715 . 24274033

[54] Chen JL, Beversdorf WD. Fatty acid inheritance in microspore-derived Populations of spring rapeseed (*Brassica napus* L.). Theoretical and Applied Genetics. 1990;80(4):465–9. 10.1007/BF00226746 . 24221003

[55] Lofton J, Godsey CB, Zhang H. Determining aluminum tolerance and critical soil pH for winter canola production for acidic soils in temperate regions. Agronomy Journal. 2010;102(1):327–32. 10.2134/agronj2009.0252

[56] Baud S, Lepiniec L. Physiological and developmental regulation of seed oil production. Progress in Lipid Research. 2010;49(3):235–49. 10.1016/j.plipres.2010.01.001 . 20102727

[57] Bonaventure G, Salas JJ, Pollard MR, Ohlrogge JB. Disruption of the FATB gene in Arabidopsis demonstrates an essential role of saturated fatty acids in plant growth. Plant Cell. 2003;15(4):1020 10.1105/tpc.008946 . 12671095 PMC152346

[58] Salas JJ, Ohlrogge JB. Characterization of substrate specificity of plant FatA and FatB acyl-ACP thioesterases. Archives of Biochemistry and Biophysics. 2002;403(1):25–34. 10.1016/S0003-9861(02)00017-6 . 12061798

[59] Okuley J, Lightner J, Feldmann K, Yadav N, Lark E, Browse J. Arabidopsis FAD2 gene encodes the enzyme that is essential for polyunsaturated lipid synthesis. Plant Cell. 1994;6(1):147 10.1105/tpc.6.1.147 . 7907506 PMC160423

[60] James DW, Lim E, Keller J, Plooy I, Ralston E, Dooner HK. Directed tagging of the Arabidopsis FATTY ACID ELONGATION1 (FAE1) gene with the maize transposon activator. Plant Cell. 1995;7(3):309–19. 10.1105/tpc.7.3.309 . 7734965 PMC160784

[61] Wang N, Wang Y, Tian F, King GJ, Zhang C, Long Y, et al A functional genomics resource for *Brassica napus*: development of an EMS mutagenized population and discovery of FAE1 point mutations by TILLING. New Phytologist. 2008;180(4):751–65. 10.1111/j.1469-8137.2008.02619.x . 18811617

[62] Rowland O, Zheng H, Hepworth SR, Lam P, Jetter R, Kunst L. CER4 encodes an alcohol-forming fatty acyl-coenzyme A reductase involved in cuticular wax production in Arabidopsis. Plant Physiology. 2006;142(3):866–77. 10.1104/pp.106.086785 16980563 PMC1630741

[63] Huang J, Xue C, Wang H, Wang L, Schmidt W, Shen R, et al Genes of ACYL CARRIER PROTEIN family show different expression profiles and overexpression of ACYL CARRIER PROTEIN 5 modulates fatty acid composition and enhances salt stress tolerance in Arabidopsis. Frontiers in Plant Science. 2017;8:987 10.3389/fpls.2017.00987 . 28642782 PMC5463277

[64] Moreno-Pérez AJ, Mónica VC, Vaistij FE, Salas JJ, Larson TR, Rafael G, et al Reduced expression of FatA thioesterases in Arabidopsis affects the oil content and fatty acid composition of the seeds. Planta. 2012;235(3):629–39. 10.1007/s00425-011-1534-5 . 22002626

