# Supplementary material for: Identification of SNP loci and candidate genes related to four important fatty acid composition in Brassica napus using genome wide association study
Source: PLoS One. 2019 Aug 23;14(8):e0221578. doi: 10.1371/journal.pone.0221578 (PMC6707581; doi:10.1371/journal.pone.0221578)
Supplement: S1 Fig — The gene expression of ACP5 (A), FAD2 (B), KCS17 (C) in diverse rapeseed accessions and tissues. (DOCX) [file pone.0221578.s001.docx]

Supplemental Fig. 1. The gene expression of *ACP5* (A), *FAD2* (B), *KCS17* (C) in diverse rapeseed accessions and tissues.


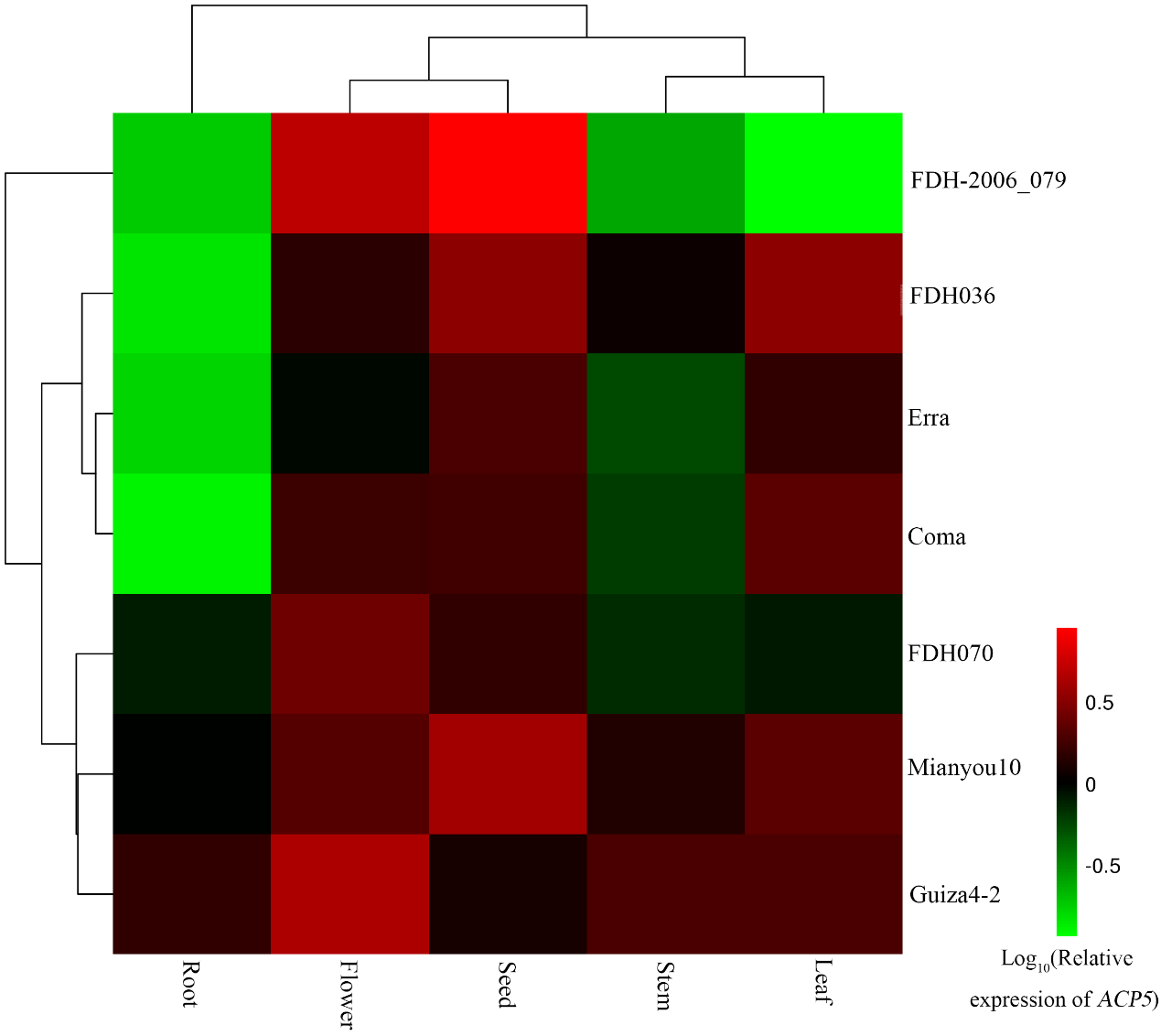


**A**


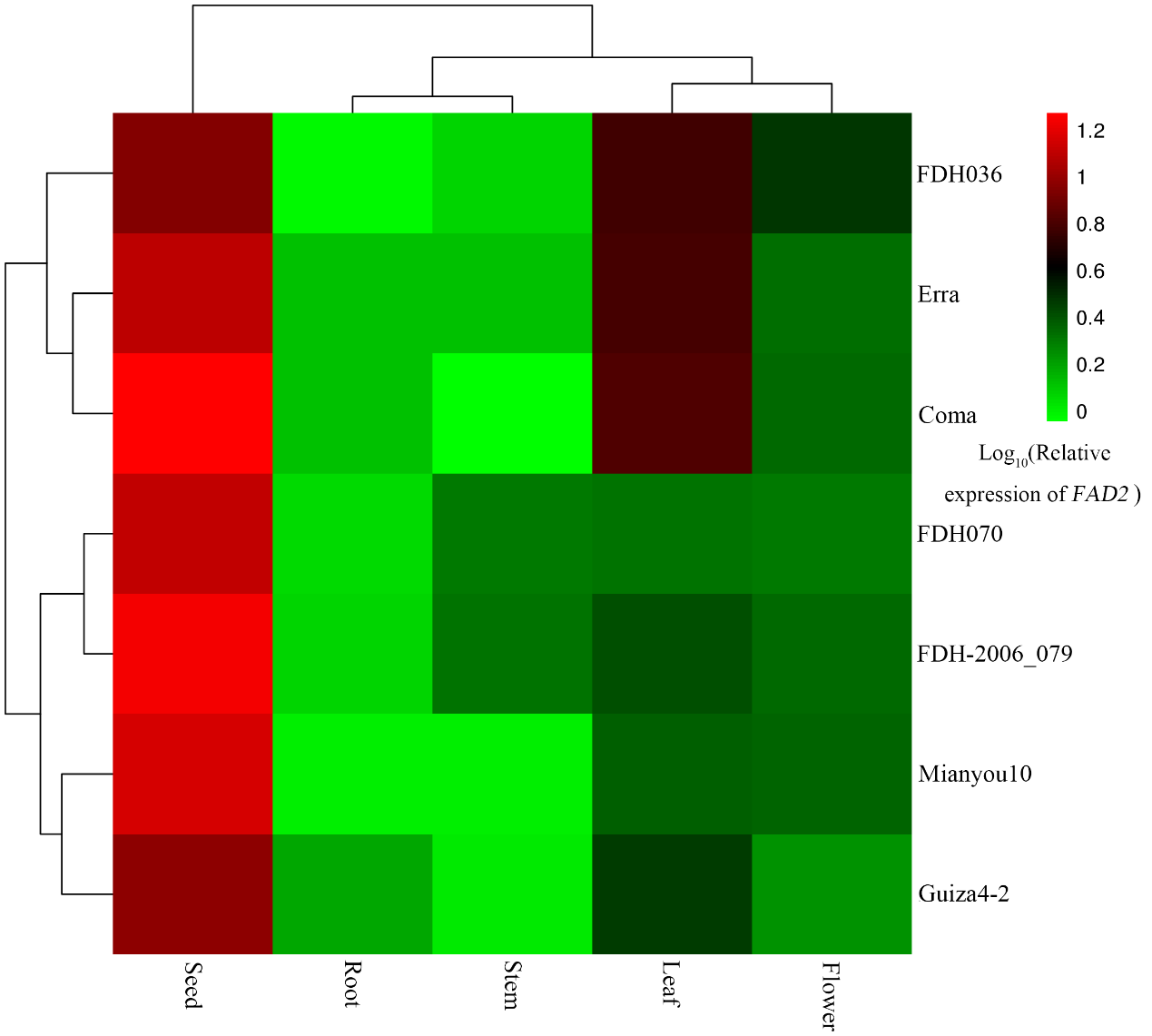


**B**


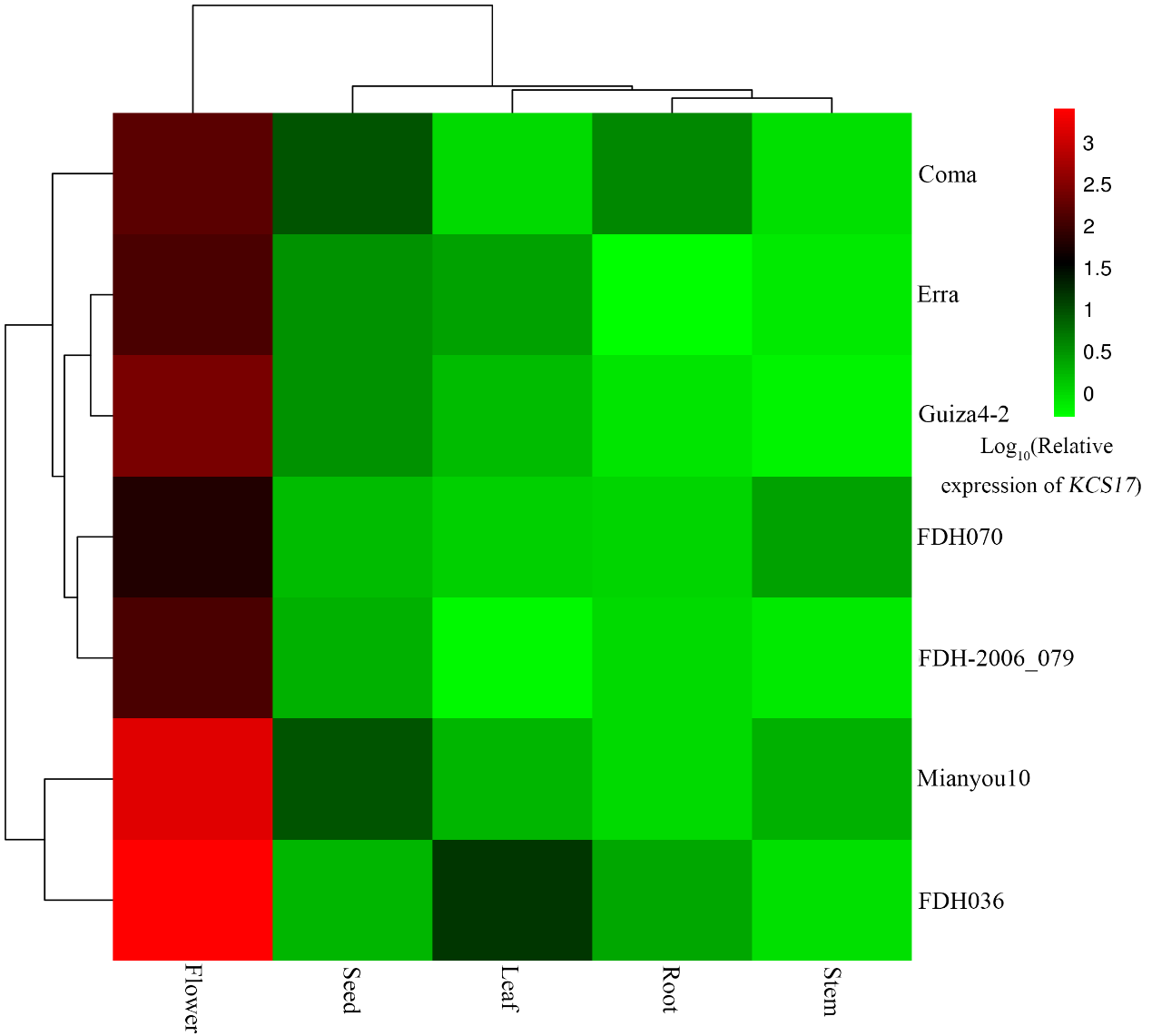


**C**
